# Comparing zero-profile and conventional cage and plate in anterior cervical discectomy and fusion using finite-element modeling

**DOI:** 10.1038/s41598-023-43086-x

**Published:** 2023-09-22

**Authors:** Chang-Hwan Ahn, Sungwook Kang, Mingoo Cho, Seong-Hun Kim, Chi Heon Kim, Inbo Han, Chul-Hyun Kim, Sung Hyun Noh, Kyoung-Tae Kim, Jong-Moon Hwang

**Affiliations:** 1 Department of Rehabilitation Medicine, Kyungpook National University Hospital, Daegu, 41944 Republic of Korea; 2 Precision Mechanical Process and Control R&D Group, Korea Institute of Industrial Technology, Jinju-si, Gyeongsangnam-do 52845 Republic of Korea; 3 Department of Neurosurgery, Seoul National University College of Medicine, Seoul, Republic of Korea; 4 Department of Neurosurgery, CHA University, School of Medicine, CHA Bundang Medical Center, Seongnam, Republic of Korea; 5 Department of Neurosurgery, Ajou University College of Medicine, Suwon, Republic of Korea; 6 Department of Neurosurgery, Kyungpook National University Hospital, Daegu, 41944 Republic of Korea; 7 Department of Neurosurgery, School of Medicine, Kyungpook National University, Daegu, 41944 Republic of Korea; 8 Department of Rehabilitation Medicine, School of Medicine, Kyungpook National University, Daegu, 41944 Republic of Korea

**Keywords:** Neuroscience, Neurology

## Abstract

Conventional cage and plate (CCP) implants usually used in ACDF surgery, do have limitations such as the development of postoperative dysphagia, adjacent segment degeneration, and soft tissue injury. To reduce the risk of these complications, zero-profile stand-alone cage were developed. We used finite-element modeling to compare the total von Mises stress applied to the bone, disc, endplate, cage and screw when using CCP and ZPSC implants. A 3-dimensional FE (Finite element) analysis was performed to investigate the effects of the CCP implant and ZPSC on the C3 ~ T1 vertebrae. We confirmed that the maximum von Mises stress applied with ZPSC implants was more than 2 times greater in the endplate than that applied with CCP implants. The 3D analysis of the ZPSC model von Mises stress measurements of screw shows areas of higher stress in red. Although using ZPSC implants in ACDF reduces CCP implant-related sequalae such as dysphagia, we have shown that greater von Mises stress is applied to the endplate, and screw when using ZPSC implants. This may explain the higher subsidence rate associated with ZPSC implant use in ACDF. When selecting an implant in ACDF, surgeons should consider patient characteristics and the advantages and disadvantages of each implant type.

## Introduction

Cervical spondylosis is a major cause of spinal cord dysfunction^1,2^. If non-surgical treatment is unsuccessful, anterior cervical discectomy and fusion (ACDF) is considered the best surgical treatment^3–6^. During ACDF, a conventional cage and plate (CCP) implant is often used, which prevents interbody graft dislocation and improves sagittal alignment, interbody fusion rate, and stability^1,5,7^. CCP implants, which are thinner than earlier models, do however have limitations such as the development of postoperative dysphagia, adjacent segment degeneration, and soft tissue injury^1,3,8–11^. While most patients recover from dysphagia by three months after surgery, 3–21% of patients do not and go on to develop chronic dysphagia^3,9,12^. To reduce the risk of these complications, zero-profile stand-alone cage implants (ZPSC; Synthes GmbH Switzerland, Oberdorf, Switzerland) were developed^8^. These implants combine an interbody pace with an anterior plate, and comprise a cage and plate with locking screws^1,3^. Several studies show that ZPSC implants reduce complications typically associated with CCP implants. Using ZPSC implants in ACDF surgery can reduce rate of postoperative dysphagia when compred to using CCP implants^13,14^. In addition to reduce the incidence of postoperative dysphagia, ZPSC implants can reduced operative time, intraoperative blood loss, and postoperate pain^1^. However there are studies that using ZPSC implants can result in a higher subsidence rate^15,16^.. Cage subsidence, a common complication of ACDF, can lead to loss of intervertebral disc height, disruption of the sagittal alignment of the spine, prevention of tight fusion, and foraminal restenosis^15,19^.

In this study, we will use finite-element modeling (FEM) to compare the total von Mises stress applied to the bone, disc, endplate, cage and screw when using CCP and ZPSC implants.

## Method

### Development of the finite element model

**Figure 1:**
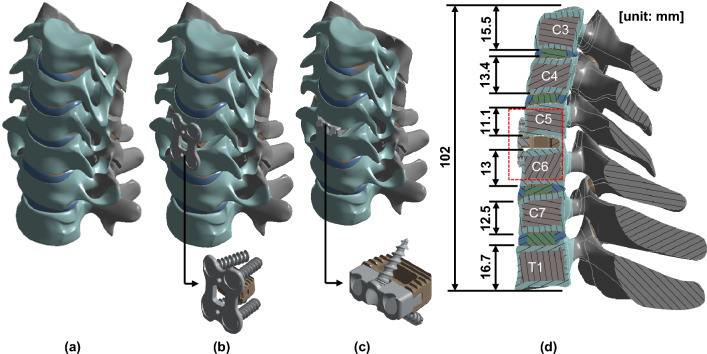
Analysis model; (**a**) Reference model, (**b**) CCP implant model, (**c**) ZPSC model, (**d**) Section view of the CCP implant model.

A 3-dimensional FEM analysis was performed under three conditions to investigate the effects on the third cervical (C3) to the first thoracic (T1) vertebrae: surgery with a CCP implant, surgery with a ZPSC implant, and reference without surgery. The model comprised C3-T1 (including cortical and cancellous bones), intervertebral discs (including annulus fibrosus and nucleus pulposus), end plates (upper and lower endplates) and posterior elements (including pedicles, laminar and facet joints). The cage system comprised bolts, plates, and artificial discs as shown in Fig. 1. The spine was modeled using data from previous studies^18,19^. The cage system was located anteriorly midway between the fifth cervical (C5) and sixth cervical (C6) vertebrae. While the CCP implant model was applied to a natural cervical spine model, the ZPSC model was applied to artificial discs. No patients were directly involved in the study. The ANSYS SpaceClaim (SpaceClaim Corporation, Concord, MA, USA) was used for modifying 3D modeling.

### Mesh and material properties for the FEM

FEM analysis was performed using the ANSYS Workbench’s Static Structural module. Due to anatomical irregularity of the model, a quadratic tetrahedron (10 nodes) was used for the mesh. After setting the mesh size for the entire model to 1 mm, von Mises stress was calculated. In this study, we approximated the load that is generated when a person lowers their head, and set elastic characteristics. Information regarding the mesh and material properties of the FEM analysis were assumed to be homogeneous and isotropic according to the published literature^20–22^ and summarized in Table 1.

**Table 1:** Mesh and material properties.

Component	Number of nodes	Number of elements	Elastic modulus (MPa)	Poisson ratio	Reference
Cortical bone	153,123	84,916	12,000	0.3	^20^
Cancellous bone	42,536	23,743	100	0.20	^20^
Posterior element	118,941	68,743	3500	0.25	^20^
End plate	30,419	14,057	600	0.3	^20^
Nucleus pulposus	10,396	5356	3.4	0.4	^20^
Annulus fibrosus	18,184	8797	500	0.45	^20^
Facet joint	3,286	1197	10.4	0.4	^20^
Cage bolt	21,952	13,669	113,800	0.342	^22,52^

### Loading and boundary conditions

**Figure 2:**
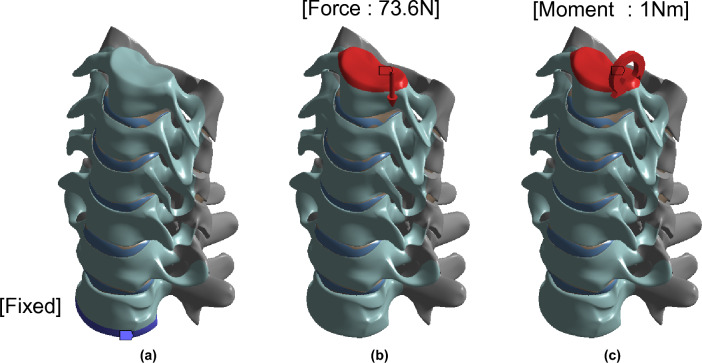
Geometry boundary and loading condition; (**a**) Fixed support, (**b**) Force condition, (**c**) Moment condition.

To examine the effect of bending the neck, the load was applied to the FEM as shown in Fig. 2. A vertical load of 73.6 N and a moment of 1.0 Nm were applied to the upper surface of the C3 cortical bone. The lower surface of the T1 cortical bone was fixed^23,24^. The cage system bolt contacted the screw via bolt thread contact (M4 × 0.7 bolt size was applied for normal plate system and M2.5 × 0.45 for the ZPSC system). We assumed the entire application surface was bonded together as one since it is extremely difficult to model the variable contact conditions of the components of the human body.

### Validation

In order to verify the validity of the finite element model used in this study, it was compared with the existing reference. Compared with the 35 cervical dimensions measured by M Nissan et al.^25^ 33 data are within the experimental data range. The remaining 2 data are within the maximum error range of 2.3 mm or less. This means that the dimensions of the finite element model presented in this study are appropriate. We also incorporate material properties based on data provided in the literature and commonly used in previous finite element models of the cervical spine to validate our model. Yoon et al.^20^ compared other experimental data for validation of FE models that measured the intersegment movement patterns of the cervical spine in flexion, extension, bilateral axial torsion and lateral bending under a pure moment of 1.0Nm. They reviewed recent FE models of the human cervical spine with a focus on the componenet modeling, material properties, and validation procedures. Their cervical spine model response was predicted under a bending moment with compressive follower loads of 50N-73N to mimic the invitro cadaver testing protocol and they showed good agreement with their experimental results. Wheel et al.^26^ provided flexion and extension data of anormal cervical spine from a young individual measuring moments ranging from 0.33Nm to 2.0Nm to the cervical spine. Wui et al.^22^ used elastic modulus = 113,000MPA and poisson's ratio = 0.342 as value for cage bolt. Tobias et al.^27^ performed an in vitro study that compared a finite element model of a human spinal sgement and twelve human cadaveric spinal segments. They revealed that the results obtained by the biomechanical analysis correlated well with the results of the finite element model. Song et al.^18^ determine the ideal amount of cement and injection site by analyzing forces with the finite element method. Tan et al.^19^ investigate the potential biomechanical factors contributing to pseudarthrosis and ASD following 3-level ACDF using a finite element cervical spine model.

## Results

**Figure 3:**
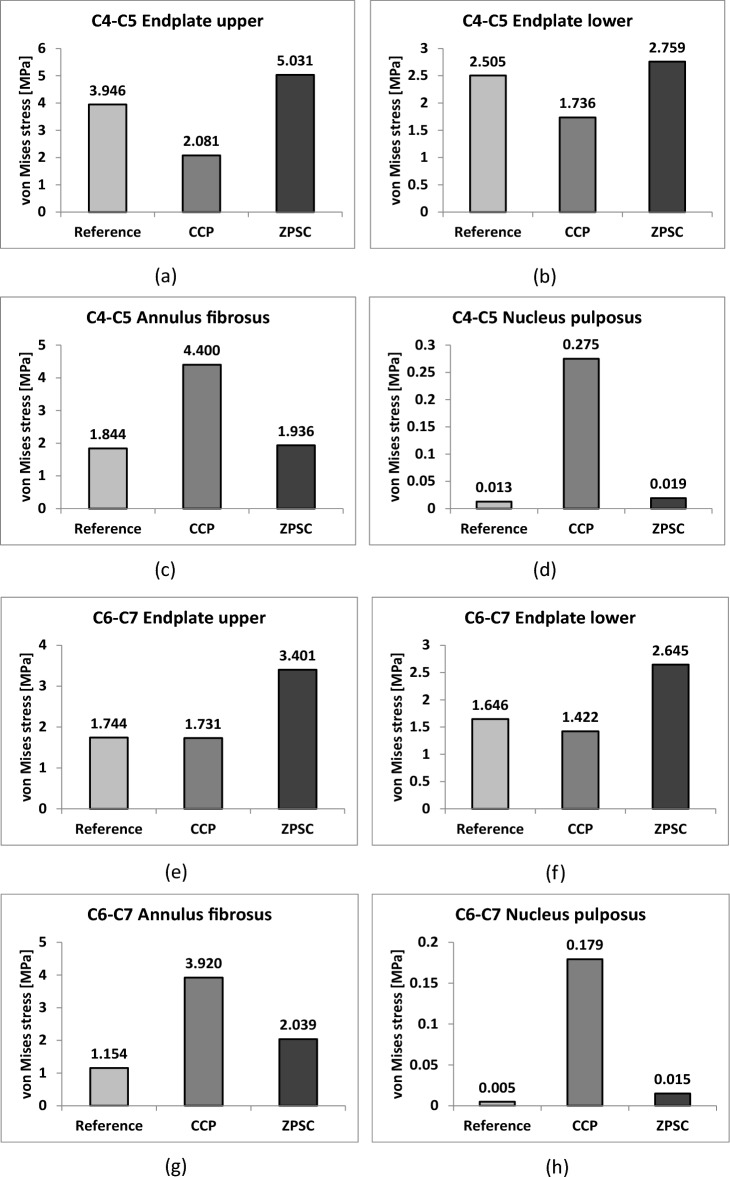

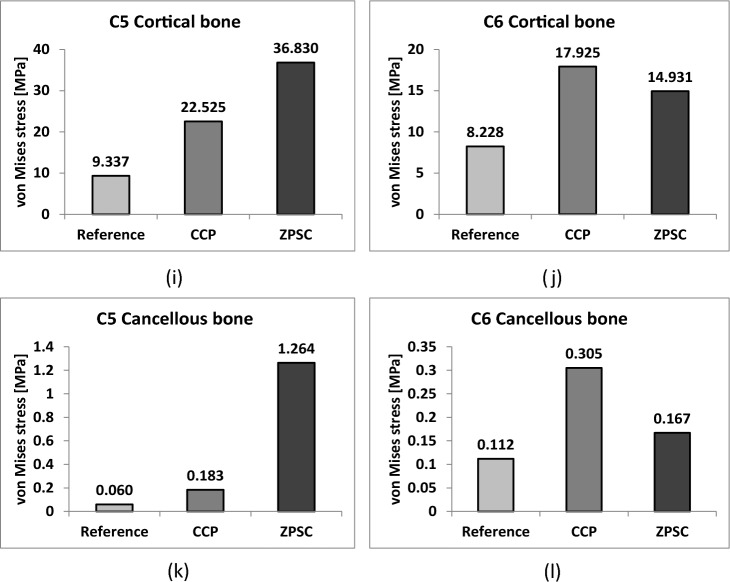
Analysis result; (**a**) C4–C5 upper endplate, (**b**) C4–C5 lower endplate, (**c**) C4–C5 annulus fibrosus, (**d**) C4–C5 nucleus pulposus, (**e**) C6–C7 upper endplate, (**f**) C6–C7 lower endplate, (**g**) C6–C7 annulus fibrosus, (**h**) C6–C7 nucleus pulposus, (**i**) C5 cortical bone, (**j**) C6 cortical bone, (**k**) C5 cancellous bone, (**l**) C6 cancellous bone.

**Figure 4:**
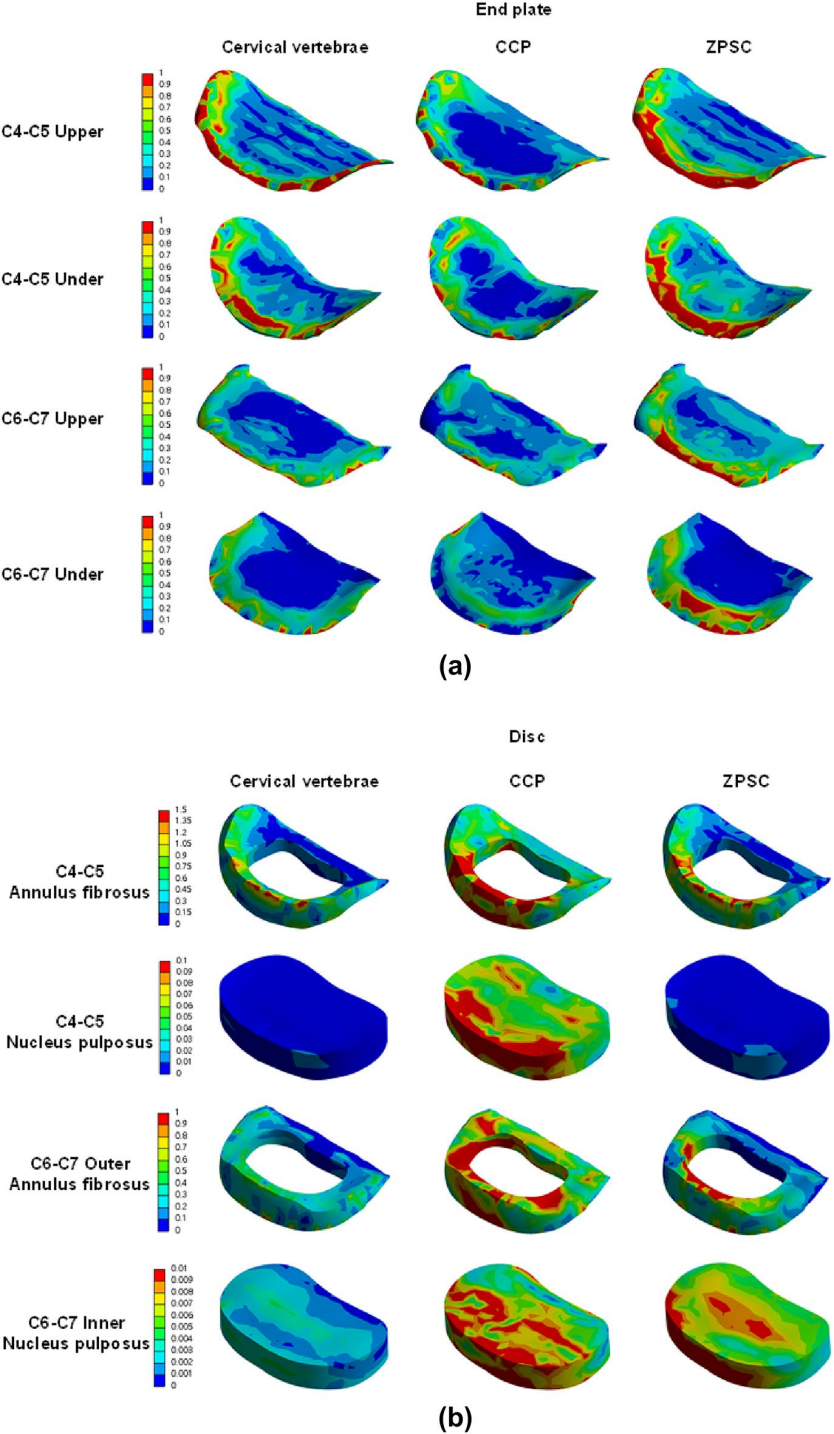

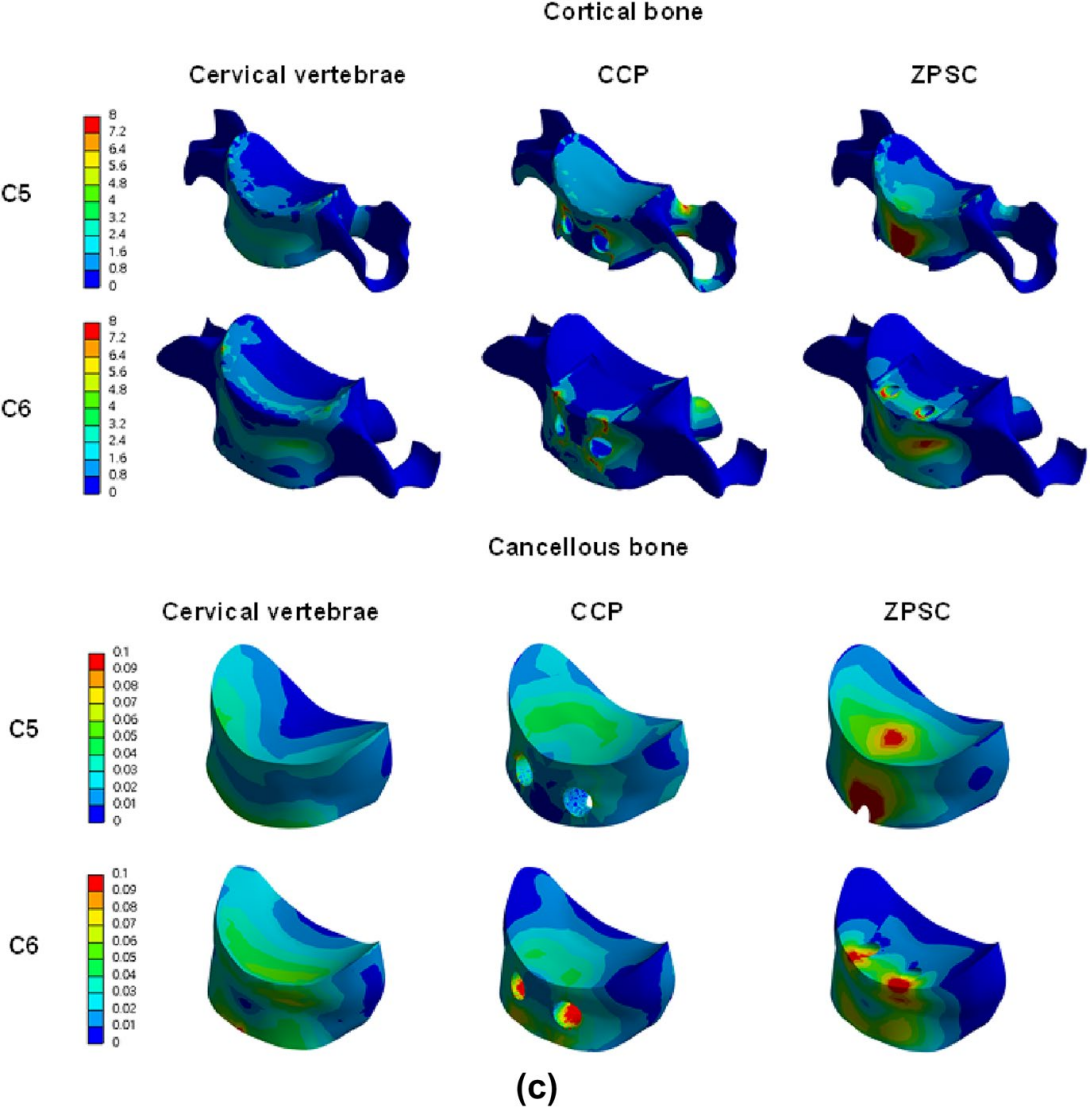
(**a**) von Mises stress results at each structure in three different models (Unit: Mpa), (**a**) upper and lower endplate, (**b**) intervertebral disc, (**c**) cortical and cancellous bone.

We measured the maximum von Mises stress applied to cortical bone, cancellous bone, the upper and lower endplate, and the intervertebral disc in three models: reference (no surgery), CCP (surgery with CCP implant), and ZPSC (surgery with ZPSC implant). Figure 3 shows the maximum von Mises stress results for each implant. Table 2 shows the contents of Fig. 2 and percentage of von Mises stress with reference for each implants. We also developed a color 3D reconstruction to illustrate the difference in von Mises stress distribution throughout the studied surface. Figure 4 show the von Mises stress distribution for the upper and lower endplate, intervertebral disc, cortical bone, and cancellous bone.

**Table 2:** von Mises stress results at each structure in three different models.

Component	von Mises stress on each model (Mpa) (% of von Mises stress with reference)		
	Reference	CCP	ZPSC
C4–C5 Endplate upper	3.946	2.081 (52.74%)	5.031 (241.76%)
C4–C5 Endplate lower	2.505	1.736 (69.30%)	2.759 (158.93%)
C4–C5 Annulus fibrosus	1.844	4.400 (238.61%)	1.936 (44.00%)
C4–C5 Nucleus pulposus	1.844	0.275 (2116.54%)	0.019 (6.98%)
C6–C7 Endplate upper	1.744	1.731 (99.25%)	3.401 (196.48%)
C6–C7 Endplate lower	1.646	1.422 (86.39%)	2.645 (186.01%)
C6–C7 Annulus fibrosus	1.154	3.920 (339.69%)	2.039 (52.02%)
C6–C7 Nucleus pulposus	0.005	0.179 (3580.00%)	0.015 (8.38%)
C5 Cortical bone	9.337	22.525 (241.24%)	36.830 (163.51%)
C6 Cortical bone	8.228	17.925 (217.85%)	14.931 (83.30%)
C5 Cancellous bone	0.060	0.183 (305.00%)	1.264 (690.71%)
C6 Cancellous bone	0.112	0.305 (272.32%)	0.167 (54.75%)
Cage		103.610	47.135
Screw		69.074	74.297

The CCP model and ZPSC model von Mises stress measurements were compared to the reference model measurements. In the C4-C5 upper endplate, lower endplate, annulus fibrosus, and nucleus pulposus, the CCP implant model measurements varied by 52.74%, 69.3%, 238.61%, and 2116.54%, respectively, while the ZPSC implant model measurements varied by 241.76%, 158.93%, 44%, and 6.98%, respectively.

In the C6-C7 upper endplate, lower endplate, annulus fibrosus, and nucleus pulposus, the CCP implant model measurements varied by 99.25%, 86.39%, 339.69%, and 3580%, respectively, while the ZPSC implant model measurements varied by 196.48%, 186.01%, 52.02%, and 8.38%, respectively.

In the C5 cortical bone and C6 cortical bone, the CCP implant model varied by 241.24%, and 163.51%, respectively. In contrast, the ZPSC model measurements decreased by 217.85%, and 83.3%, respectively.

In the C5 cancellous bone and C6 cancellous bone, the CCP implant model measurements varied by 305%, and 272.32%, respectively. The ZPSC model measurements varied by 690.71%, and 54.75%, respectively.

Because of comparing C4-C5 endplate and discs, the CCP implant model increased by an average of 4.86% compared to the reference model, and the ZPSC model increased by an average of 16.96% compared to the reference model. Because of comparing C6-C7 endplate and discs, the CCP implant model increased by an average of 37.8% compared to the reference model, and the ZPSC model increased by an average of 49.14% compared to the reference model. In the case of C5 (cortical bone and cancellous bone), the CCP implant model increased by an average of 62.92% compared to the reference model, and the ZPSC model decreased by an average of 84.95% compared to the reference model. For C6 (cortical bone and cancellous bone), the CCP implant model increased by an average of 58.68% compared to the reference model, and the ZPSC model decreased by an average of 38.88% compared to the reference model.

The maximum von Mises stress in all studied locations was greatest in the ZPSC model followed by the reference model and lastly the CCP model. There was a significant difference in the maximum von Mises stress between the reference model and the CCP implant model, and between the reference model and the ZPSC model.

The 3D analysis of the ZPSC model von Mises stress measurements shows areas of higher stress in red.

**Figure 5:**
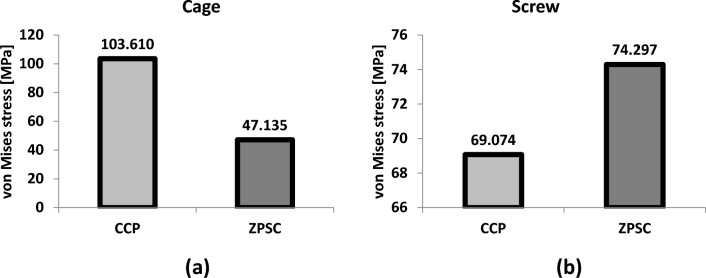
Analysis Result; (**a**) Cage, (**b**) Screw.

**Figure 6:**
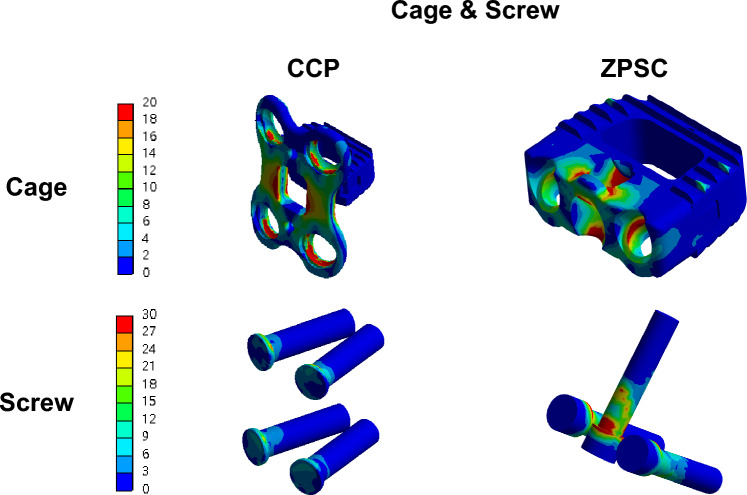
Von mises stress results at cage and screw in two different models (Unit: MPa).

We also compared the maximum von Mises stress and von Mises stress distribution of the CCP model and the ZPSC model when applied to the cage and screw in Fig. 5 and 6.

In the Cage, Von Mises stress in the ZPSC model relative to the CCP model was 45% respectively. In the 3D model of the cage in CCP model, the high areas of stress in red is distributed on the anterior plate. In case of ZPSC model, the high areas of stress in red is distributed on the cage itself. In the Screw, Von Mises stress in ZPSC model relative to the CCP model was 108% respectively. The 3D model of the screw shows high areas of stress in red for the ZPSC model.

## Discussion

Since ACDF surgeries with CCP implants were found to have a higher rate of postoperative complications such as dysphagia and soft tissue damage, ZPSC implants were developed^3,28,29^. ZPSC implant use achieved similar rates of fusion and biomechanical stability with a lower incidence of dysphagia^3,30,31^. However, ZPSC implants were found to suffer from a high subsidence rate (mean 21.1%, range 0–83%), which can increase the rate of reoperation^32,33^. Several previous studies also compared CCP implants and ZPSC implants. Haiyu et al.^1^ found ZPSC implants had a lower rate of postoperative dysphagia at 2 weeks, 6 months, and 1 year (p = 0.002, p = 0.008, and p = 0.001, respectively) than CCP implants. Alfate et al.^34^ found ZPSC implants had a lower rate of operative time, intraoperative blood loss, risk of postoperative dysphagia (p < 0.0001, respectively) than CCP implants. They also compared cage subsidence rate and there was no significant heterogeneity (P = 0.5). However Zhe et al.^15^ revealed that the overall subsidence rate was higher in the ZPSC implants than in the CCP implants (66.67% vs 38.46%, p = 0.006). In a meta-analysis conducted by Yingjje^35^ ZPSC implants had a higher incidence of postoperative subsidence than that in the CCP implants (15.1% vs 8.8%, p = 0.0005). Our study confirmed prior findings that using a ZPSC implant without a plate leads to a higher subsidence rate, interfering with disc stability and potentially incurring a higher reoperation rate^5,36,37^.

We confirmed that the maximum von Mises stress applied with ZPSC implants was more than 2 times greater in endplate and more than 2 times lower in intervertebral disc than that applied with CCP. The difference in stress application may be due to the unique structural characteristics of ZPSC implants. During ACDF surgery the 2 screws in ZPSC implants directly penetrates the endplate potentially causing cracks and compared with the CCP implants, more upper end plates should be removed in the ZPSC impalnts to make the cage match the intervertebral space^1,38^. Compromising the endplate, the main vertebral nutrient supply network, can cause degeneration of the cervical spine and higher cage subsidence^39,40^. This complication rate is exacerbated in patients with osteoporosis since vertebral load is higher in these patients^41,42^.

Moreover, the absence of the anterior plate can affect subsidence incidence. In the case of CCP implants, cage is placed within the intervertebral space with a plate in front and 2 screws locate at the superior and inferior vertebral body. However, in the case of ZPSC implants, cage is anchored by only 2 screws without a anterior plate. 2 screws are inserted through the cage and located at the same plane. The anterior plate has screws rigidly affixed to the plate and act as fixed moment arm cantilever beams providing ventral distraction fixation with the spine^43^. Therefore, if there is no anterior plate, the load is unevenly distributed leading to more von Mises stress on the bone, cage and screw^36,37^. In addition, due to the placement of the 2 screws and cage of the ZPSC implants, the cage placed in front of the intervertebral space can act as the fulcrum of the mechanics. This may produce a forward traction to the superior and inferior vertebrae and can increase the incidence of cage subsidence^44^.

Several previous studies have explored subsidence risk factors. The most common surgical technique and instrument-related risk factors include an inappropriate cage size and location, inappropriate endplate preparation, use of a cage without an anterior plate, and the use of large grafts^45,46^. Patient-related risk factors include older age and baseline cervical kyphosis^36^. We suggest that surgeons should consider using CCP implants rather than ZPSC implants when operating on patients with cervical kyphosis, osteoporosis and those aged 60 years or older. In contrast, since several studies have reported a higher incidence of dysphagia when using an anterior cervical plate or when performing multi-level surgery, it may be preferable to use ZPSC in these cases^47,48^.

There are several limitations in this study. First, although degenerative disc changes are associated with changes in material properties such as nucleus pulposus water content, we performed FEM on vertebrae assuming fixed properties since the changes with degenerative disc disease are not easily predictable. More, each patient's screw diameter may be different and insufficient length of screw can cause greater stress loads^49^. As same reason above, we performed FEM on screw assuming fixed diamter. Second, the 3D-FEM did not fully incorporate all spine structures, such as ligaments and muscles, and may not replicate actual spine biomechanics. Third, only C5-C6 ACDF surgery was studied because this level is reported to be the most commonly affected level in cervical spondylosis^50,51^, and only flexion position was included in the results. Future studies that includes other single-level ACDF surgery and multi-level ACDF surgery including other position (flexion, extension, bending, rotation) may shed further light on this discussion.

## Conclusion

Using using ZPSC implants in ACDF reduces CCP implant-related sequalae such as dysphagia. However we have shown that greater von Mises stress is applied to the endplate and screw and von Mises stress is unevenly distributed in the cage when using ZPSC implants. This may explain the higher subsidence rate associated with ZPSC implant use in ACDF. If ACDF surgery is performed using ZPSC implants considering only the disadvantages of CCP implants, sequalae such as subsidence may occur frequently. Therefore, when selecting an implant in ACDF, surgeons should consider patient characteristics and the advantages and disadvantages of each implant type.

## Data Availability

The datasets analyzed during the current study are available from the corresponding author on reasonable request.
